# High spatial resolution elevation change dataset derived from ICESat-2 crossover points on the Tibetan Plateau

**DOI:** 10.1038/s41597-024-03214-2

**Published:** 2024-04-17

**Authors:** Tengfei Chen, Jian Wang, Tao Che, Xiaohua Hao, Hongyi Li

**Affiliations:** 1 Key Laboratory of Cryospheric Science and Frozen Soil Engineering, Northwest Institute of Eco-Environment and Resources, Chinese Academy of Sciences, Lanzhou, 730000 China; 2 Faculty of Geomatics, Lanzhou Jiaotong University, Lanzhou, 730000 China; 3 National-Local Joint Engineering Research Center of Technologies and Applications for National Geo-graphic State Monitoring, Lanzhou, 730000 China; 4 Gansu Provincial Engineering Laboratory for National Geographic State Monitoring, Lanzhou, 730000 China; 5 Heihe Remote Sensing Experimental Research Station, Key Laboratory of Remote Sensing of Gansu Province, Chinese Academy of Sciences, Lanzhou, 730000 China

**Keywords:** Geomorphology, Geography

## Abstract

Understanding elevation changes on the Tibetan Plateau is crucial to comprehend the changes in topography, landscape, climate, environmental conditions, and water resources. However, some of the current products that track elevation changes only cover specific surface types or limited areas, and others have low spatial resolution. We propose an algorithm to extract ICESat-2 crossover points dataset for the Tibetan Plateau, and form a dataset. The crossover points dataset has a density of 2.015 groups/km², and each group of crossover points indicates the amount of change in elevation before and after a period of time over an area of approximately 17 meters in diameter. Comparing ICESat-2 crossover points data with existing studies on glaciers and lakes, we demonstrated the reliability of the derived elevation changes. The ICESat-2 crossover points provide a refined data source for understanding high-spatial-resolution elevation changes on the Tibetan Plateau. This dataset can provide validation data for various studies that require high-precision or high-resolution elevation change data on the Tibetan Plateau.

## Background & Summary

Surface elevation changes on the Tibetan Plateau are important indicators for studying the geography, environment, and climate of the region. These changes can be attributed to various causes. Lithospheric deformation causes slow surface changes, while natural disasters can lead to more dramatic changes^1,2^. Additionally, surface elevation changes can occur due to the impact of climate change on snow mountains, glaciers, permafrost, and snow^3^.

High spatial resolution elevation change data is essential for several reasons. Firstly, it allows for the capture of smaller-scale terrain changes, such as those triggered by natural disasters like mudslides and landslides. This data helps us understand surface deformation in inaccessible areas caused by these disasters. Secondly, high spatial resolution surface elevation change data provides a more detailed quantitative analysis of surface elevation change. For instance, it can offer permafrost elevation changes in small areas, which can serve as a reference for engineering and construction safety in cold regions. Lastly, high spatial resolution surface elevation change data can serve as basic data support for other studies. It can validate model simulation results or refine existing large-scale products as supplementary data.

However, high spatial resolution data on surface elevation changes is lacking. Existing data on elevation changes in the Tibetan Plateau are either specific to individual areas rather than the entire plateau or have low spatial resolution. For example, a lake-level dataset generated from multi-source altimetry satellite data and Landsat optical imagery covers the water level changes of 52 large and medium-sized lakes on the Qinghai-Tibetan Plateau^3^. Another lake-level dataset, which integrates eight laser altimeter data such as Envisat, ICESat, and CryoSat-2, covers lakes larger than 10 km^2^ on the Tibetan Plateau^4^. These provide lake water level elevations for the entire lake. A glacier change dataset generated from SRTM 2000 and ASTER stereo pairs before and after 2015 estimates elevation changes in over 40 typical glacier regions on the Tibetan Plateau^5^. Among satellite altimetry data, ICESat-2 data have high accuracy (about 0.1 m), a small spatial footprint (17 m), and a very close distance between laser footprints along the orbital direction (20 m)^6^. However, ICESat-2 track revisit positions are not fixed, and points of the same track are not exactly repeated in different cycles^7^.

A comprehensive ICESat-2 crossover points dataset, encompassing all terrains, is necessary. While ICESat-2 can provide precise surface elevation data, we often lack the specific elevation before any surface changes. This issue presents a challenge in capturing the surface elevation change within certain areas. Many researchers have utilized the complete ICESat-2 data to analyze surface elevation changes^8–10^. However, it’s not suitable to simply fit the data from an identical time point over a vast area to a single plane in complex terrain environments. This method could overlook critical details of surface deformation. Furthermore, with the evolution of various models for the Tibetan Plateau (including hydrological, glacier, ecological and vegetation models)^11–13^, there is an increasing demand for high spatial resolution surface elevation changes to be used as training data. Therefore, several scholars have used the ICESat-2 crossover points method in their studies. For example, one study on the rate of descent of the Svalbard Archipelago ice sheet utilized the crossover points method. In this study, the authors used ICESat-1 and ICESat-2 to extract crossover points, interpolated the elevation change at those points, and finally calculated the ice sheet’s decline rate^14^. However, due to the long distance of 170 m between neighbouring footprints along the track direction of ICESat-1, the number of crossover points is low. Another study examined the accuracy of ICESat-2 in shallow water and referred to using crossover points to obtain elevation changes. And, the authors illustrated the higher accuracy of the results obtained at the crossover points of the ICESat-2 strong beams^15^. Additionally, the effectiveness of ICESat-2 crossover points for snow depth identification has been evaluated in flat areas^16^. Although the crossover points method can resolve surface variations associated with changes in snow depth and seasonal melting of the active permafrost layer, its accuracy is affected by the slope and roughness of the terrain^17^.

Specifically, (1) existing datasets obtained based on reanalysis of ICESat-2, although using the crossover points method, only cover glaciers and lakes, not the wider surface types of the Tibetan Plateau, such as permafrost, vegetation, deserts, and others. (2) Existing studies lack a uniform definition standard for ICESat-2 crossover points, a clear description of crossover points methods, and a more detailed description of extraction algorithms. (3) The remote location of the Tibetan Plateau poses challenges for researchers in collecting accurate data on surface deformation. Consequently, many studies lack the required validation data to accurately measure elevation changes.

To address these issues, we produced the ICESat-2 crossover points dataset for the entire Tibetan Plateau, covering the period from September 2018 to October 2022. We calculated the accuracy of the elevation differences provided by each group of crossover points using existing ICESat-2 validation results on the plateau. To assess its reliability, we compared the crossover points dataset with existing studies on glaciers and lakes on the Tibetan Plateau. This experiment introduces the definition criteria of crossover points and shows the extraction algorithm, distinguishing it from previous studies. Furthermore, the crossover point dataset extracted in this experiment covers most of the Tibetan Plateau, including all types of terrain and slope, providing a reference for studying surface elevation changes on the plateau. It offers high-resolution surface elevation change information at crossover point locations. The ICESat-2 crossover points dataset has a wide range of applications. For instance, it can be used as research data on changes in the permafrost layer of the Tibetan Plateau, as supplementary data for existing glacials and lakes studies, or combined with multi-source remote sensing data to form surface elevation changes on a continuous time series. It can also serve as validation data for various types of topographic studies.

**Fig. 1:**
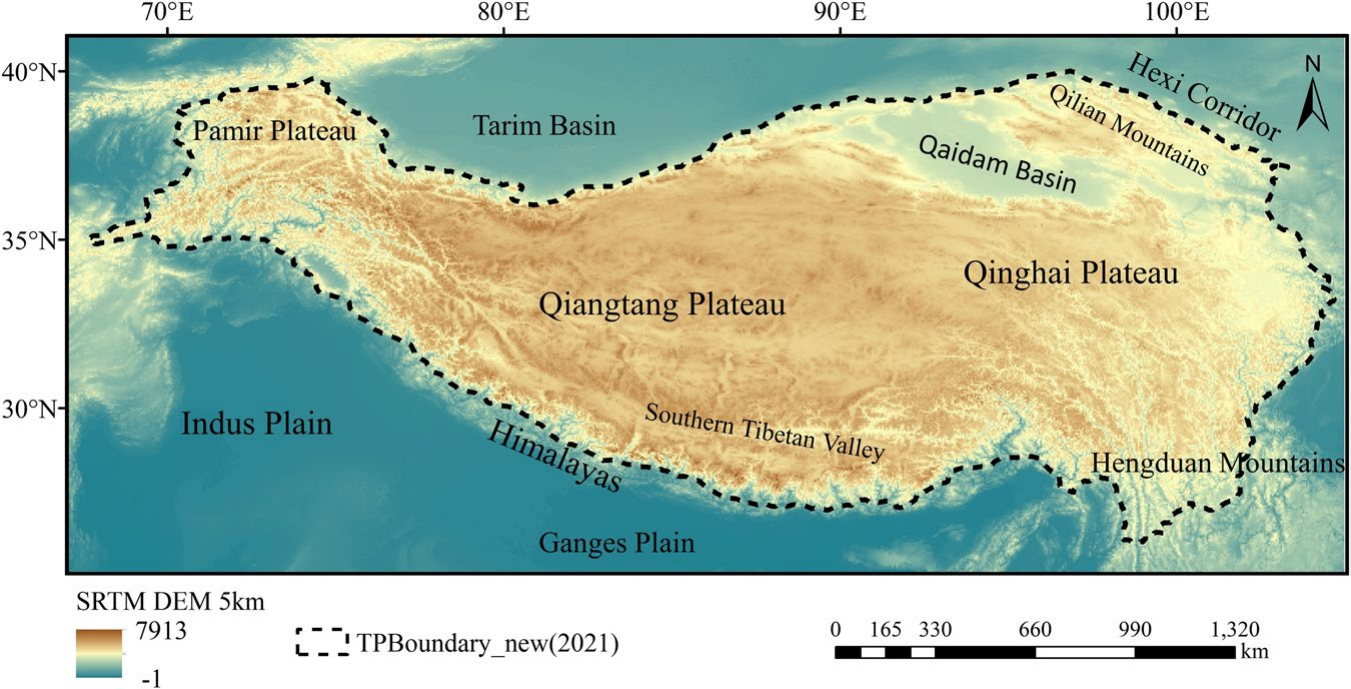
Study area. The black dashed line in the figure represents the boundary of the Tibetan Plateau^51^. The background image is a 5 km SRTM DEM. The figure also includes an indication of some of the terrain within and around the Tibetan Plateau.

## Methods

### Study area

The Tibetan Plateau, situated in the southern part of Asia (67° E-105° E, 25° N-41° N), features a continental climate with strong radiation and substantial temperature differences^18^. It boasts an average elevation exceeding 4,000 m, with the lowest point at 900 m and the highest peak reaching 8,844 m^19^. The plateau encompasses various regions including the Qiangtang Plateau, the Southern Tibetan Valley, the Qaidam Basin, the Qilian Mountains, the Qinghai Plateau, and the Sichuan-Tibet alpine canyon area (Hengduan Mountains) (Fig. 1). Additionally, it includes diverse landscapes such as permafrost, glaciers, grasslands, deserts, and lakes, contributing to its complex and undulating terrain.Apart from the North and South Poles, the Tibetan Plateau is the most extensively glaciated region on earth^20^. It has the highest and largest area of alpine and plateau permafrost in the mid-latitudes of the Northern Hemisphere^21^. The plateau is also densely covered with lakes, glaciers, frozen soils, swamps, and wetlands, which together store a significant amount of the world’s water resources. In recent years, global environmental changes significantly impacted water resources, such as glaciers, permafrost, and lakes, leading to rapid changes in the surface elevation of the Tibetan Plateau. These changes have a broader impact on the surrounding countries and even global climate change^22^.

### Data sources

#### ICESat-2^23^

ICESat-2 was launched on September 15, 2018, equipped with the Advanced Topographic Laser Altimeter System (ATLAS). ATLAS captures elevation data in six tracks, emitting photons of different intensities, divided into three strong and three weak tracks. The ICESat-2 footprint measures 17 meters in diameter and has a repetition period of 91 days. NASA has published 21 data products from ATL00 to ATL21 for different research objectives, categorized into four data levels. For this experiment, we utilized the Level 3 land ice height product ICESat-2 ATL06 and the Level 3 land and vegetation height product ICESat-2 ATL08, which includes parameters such as latitude, longitude, time, elevation value, and quality control parameters (Table 1).

**Table 1:** ICESat-2 products and data used in comparative experiments.

Data	Time	Data sources	Spatial resolution	Data role	Data references
ICESat-2 ATL06	2018.09-2022.10	NASA	17 m	Extracting ICESat-2 crossover points	Smith *et al*., 2023
ICESat-2 ATL08	2018.09-2022.10	NASA	100 m	Extracting ICESat-2 crossover points	Smith *et al*., 2023
UAV Data	2023.03	DJI L1 LIDAR	0.5 m	Data validation	This study
Glacier Boundary	2017	Glacier coverage data on the Tibetan Plateau in 2017 (TPG2017, Versionl.0)	30 m	Screening of glacial ICESat-2 Crossover Points	Ye, 2019
Lake Boundary	2020	The lakes larger than 1 km² in Tibetan Plateau (V3.1) (1970s-2022)	10 m-100 m	Screening lakes ICESat-2 crossing points	Zhang, 2022

#### UAV (Unmanned Aerial Vehicle) Data

We conducted an observational experiment using a UAV in the Qilian Mountains region, located in the northeastern Tibetan Plateau. The UAV was equipped with DJI L1 LIDAR. The observations were made in March 2023. The initial data for drone construction was a laser point cloud. This data was first denoised, then the ground points were extracted, and finally, it was converted into a 0.5 m DEM. We used this data to validate the accuracy of the ATL08 and ATL06 products from ICESat-2 (Table 1).

#### Tibetan Plateau Glacier Data^24,25^

The “Glacier coverage data on the Tibetan Plateau in 2017 (TPG2017, Version1.0)” dataset published on the Spatio-Temporal Tertiary Environmental Big Data Platform is the source of the glacier data on the Tibetan Plateau. The dataset used 210 Landsat8 OLI multispectral remote sensing images from 2013 to 2018. It was generated using visual interpretation, with Landsat8 OLI data from 2017 accounting for 90 percent of the total data. 85 percent of the Landsat8 OLI data was imaged in winter. The dataset provides a glacier raster resolution of 30 m. We used the data to filter ICESat-2 crossover points on the glacier and compared the filtered ICESat-2 crossover points with existing studies on glaciers on the Tibetan Plateau (Table 1).

#### Tibetan Plateau Lake Data^26–28^

The “The lakes larger than 1 km² in Tibetan Plateau (v3.1) (1970s-2022)” published on the Spatial and Temporal Tertiary Environmental Big Data Platform is the source of the lake data on the Tibetan Plateau. The dataset used long-time Landsat remote sensing data to obtain lake observations for 16 phases over nearly 50 years from the 1970s to 2022 on the Tibetan Plateau. The dataset counted the number and area of lakes larger than 1 km² and provided year-by-year lake ranges. The lake year chosen for this experiment is 2020. We used the data to filter ICESat-2 crossover points (Table 1).

### Determining ICESat-2 crossover points

**Fig. 2:**
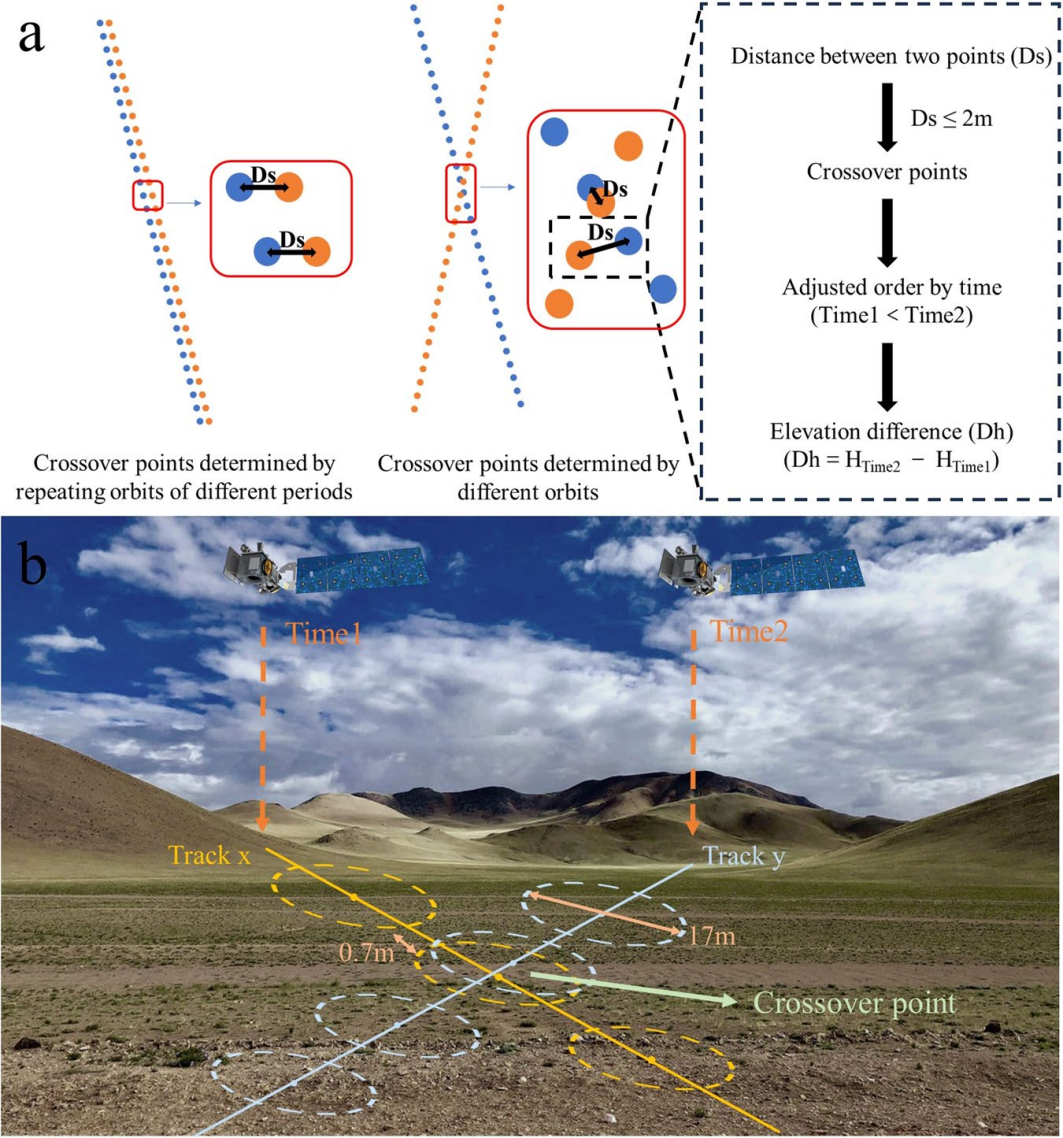
Definition of Crossover Points. The left figure in Fig. (**a**) shows how crossover points are determined using ICESat-2 data from two different times, represented by blue and orange points. The right figure of Fig. (**a**) shows the determination criterion for the crossover points and the definition of the elevation difference. Fig. (**b**) is a schematic representation of ICESat-2 crossover points determined by different tracks. The solid blue and yellow lines represent the two ICESat-2 tracks, Time1 and Time2 indicate the acquisition times, and the dashed circles indicate the laser footprints.

Crossover points of ICESat-2 are determined in two ways (Fig. 2a):

1. ICESat-2 has 1387 tracks. In the same track, ICESat-2 of different cycles may overlap. In this experiment, these repetition points are called intersections.
2. Crossover points are extracted at locations where different orbits intersect. Additionally, this experiment sets a distance limit between two points in the crossover points. If the distance between the centers of two ICESat-2 laser footprints is less than 2 m, these two points form a group of crossover points.

**Fig. 3:**
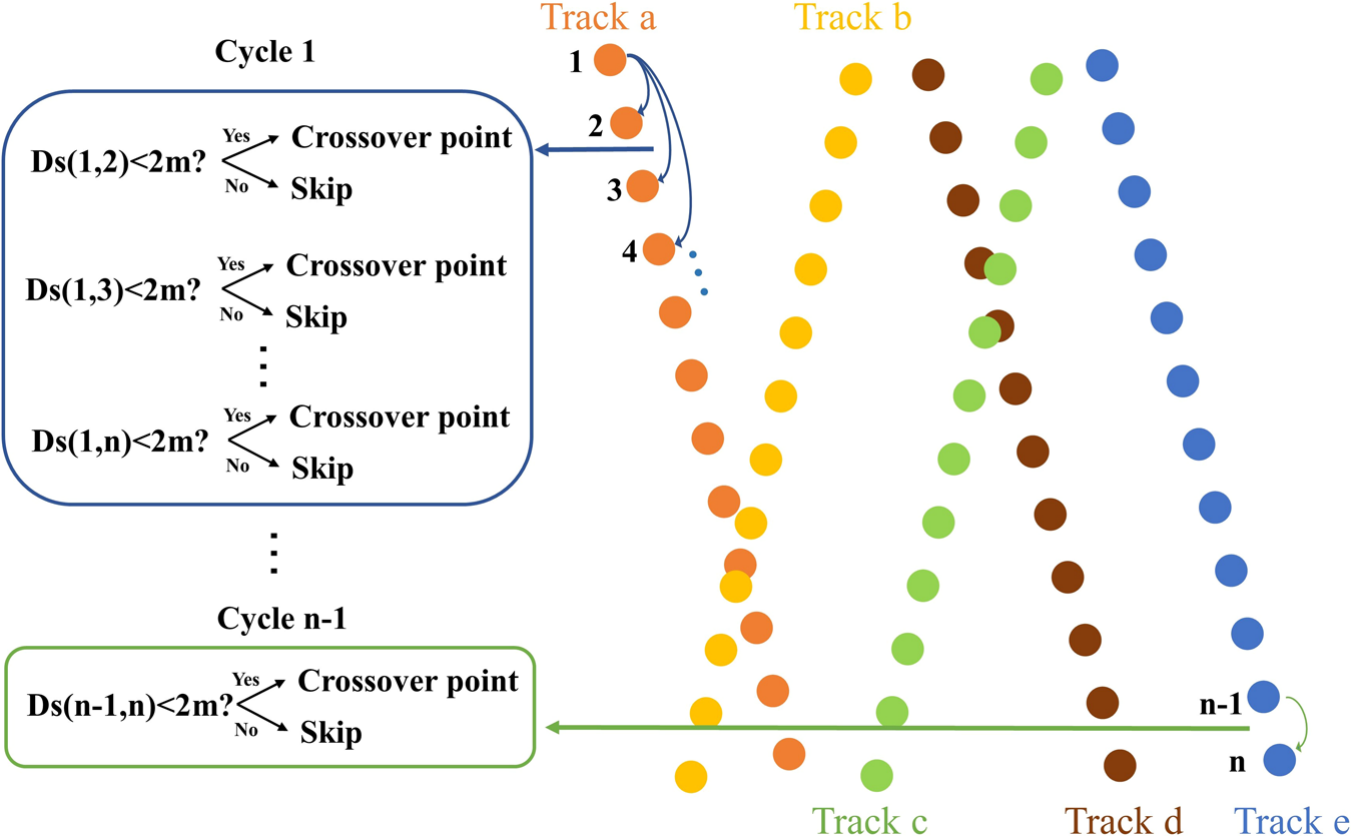
Algorithm for extracting ICESat-2 crossover points. The right half of the diagram displays all ICESat-2 points within a region. The different colored points represent ICESat-2 points on different tracks. We assign a specific serial number to each point (point 1 to point n). The left half of the diagram shows the discriminatory and cyclical process for identifying the crossover points. n points require n-1 cycles. For Cycle 1, we calculate the distance between the first point and each of the remaining points to determine the crossover points.

Figure 2b provides general features of ICESat-2. The overlap between the two footprints of each group of ICESat-2 crossover points is high (Fig. 2b), allowing for more accurate elevation change measurements. There are several loops and iterations to extract crossover points from all ICESat-2 points (Fig. 3). First, we identified a set of all ICESat-2 points in a region from September 2018 to October 2022 and assigned a specific ordinal number to each point (assuming a total of *n* ICESat-2 points).

In the first loop, we calculated the distance between point 1 and the remaining points in this set (point 2 to point *n*) one by one. If the distance between two points was less than 2 m, we considered them a group of ICESat-2 crossover points. After the first loop, we started the second loop. We excluded point 1 and then calculated the distance between point 2 and the remaining points in this set (point 3 to point *n*) to determine all crossover points. We applied a similar method to identify crossover points between point 3 and point n-1 within each loop. We combined all the crossover points within each loop to obtain the ICESat-2 crossover points data for the region.

To calculate the distance between two points, we used the Haversine formula^29^. In ICESat-2 ATL06 and ATL08, NASA provides the latitude and longitude of each point. The Haversine formula is a method of calculating the distance between two points using their latitude and longitude. The equation for the Haversine formula is as follows.

1 $${\varphi }_{1}=\frac{{{\rm{lat}}}_{{\rm{A}}}\times \pi }{180},\,{\varphi }_{2}=\frac{{{\rm{lat}}}_{{\rm{B}}}\times \pi }{180}$$

2 $${\theta }_{1}=\frac{{{\rm{lon}}}_{{\rm{A}}}\times \pi }{180},\,{{\rm{\theta }}}_{2}=\frac{{{\rm{lon}}}_{{\rm{B}}}\times \pi }{180}$$

3 $${\rm{d}}=2{\rm{r}}\times \arcsin \left(\sqrt{{\sin }^{2}\left(\frac{{\varphi }_{1}-{\varphi }_{2}}{2}\right)+\cos \,{\varphi }_{1}\times \cos \,{\varphi }_{2}\times {\sin }^{2}\left(\frac{{\theta }_{1}-{\theta }_{2}}{2}\right)}\right)$$

In the equation, A and B represent the two ICESat-2 points. latA and latB represent the latitude of points A and B, respectively, while lonA and lonB represent their respective longitudes. *φ*1 and *φ*2 represent the radian regime measures of latitude of points A and B, respectively, while θ1 and θ2 represent their respective radian regime measures of longitude. d represents the actual distance between A and B, and r represents the radius of the Earth. Furthermore, we arranged the two points within each group of crossover points in chronological order and calculated the elevation difference using Eq. 4.

4 $${{\rm{DH}}}_{{\rm{ICESat}}}-2={\rm{H}}\_{\rm{Time}}2-{\rm{H}}\_{\rm{Time}}1$$

In the equation, DH_ICESat–2 represents the elevation difference at each group of crossover points.H_Time2 and H_Time1 represent the elevation of two points in each group of crossover points, respectively, with Time1 being the earlier time.

To obtain all ICESat-2 crossover points over the entire Tibetan Plateau, we first performed data filtering and partitioning on the original ICESat-2 data. We also removed outliers from all crossover points.

### Filtering and Partitioning of ICESat-2 Data over the Tibetan Plateau

We followed a two-step process to filter the data before extracting ICESat-2 crossover points.

First, we filtered the strong and weak photons. ICESat-2 acquires elevation data with six beams, which are divided into three weak and three strong beams. Previous studies have shown that the strong beam photons lose less energy in propagation, have a wider range of applications, and are more accurate^30,31^. Therefore, we chose to use information only from the photons of the three strong beams in this experiment.

Second, we filtered out the faulty photons. ICESat-2 ATL06 provides a laser point elevation quality filter parameter, “atl06_quality_summary.” The data label for faulty photons is 1, while it is 0 for accurate photons. We removed the faulty photons by filtering out all data labelled with 0.

We partitioned the ICESat-2 data based on spatial location. The number of ATL06 and ATL08 points on the Tibetan Plateau from 2018 to 2022 is very large, making it difficult to run the crossover points algorithm for all ICESat-2 laser points. In this experiment, we divided the Tibetan Plateau into 5051 small regions at 0.25° intervals for latitude and longitude and extracted the crossover points of ICESat-2 for each region. By combining the extraction results from all regions, we obtained the ICESat-2 crossover points dataset for the Tibetan Plateau.

### Removing outliers from ICESat-2 crossover points

Outliers in the elevation difference at ICESat-2 crossover points can be caused by factors such as cloud cover and complex terrain. These outliers can be identified and removed using the 3σ criterion. If the difference between the elevation difference at the crossover points and the mean elevation difference is more than three times the standard deviation, it is deemed an outlier.

The average elevation difference at all ICESat-2 ATL06 crossover points is −0.052 m with a standard deviation of 3.314 m. After outlier removal, the average becomes −0.040 m with a standard deviation of 1.369 m. For ICESat-2 ATL08 crossover points, the average elevation difference is −0.301 m with a standard deviation of 5.767 m. After outlier removal, these values change to an average of −0.260 m and a standard deviation of 3.178 m.

### Statistics on surrounding points

Crossover points may cluster at the same location, providing repeated surface elevation data. To identify these points accurately, we first establish a 4 m buffer around each point. Then, we count the number of points within each buffer. We chose a 4 m buffer to ensure we capture enough neighboring crossover points. Finally, we record the total number of surrounding points in the “Around_PT” field for each point. If “Around_PT” equals 2, it signifies that only one group of ICESat-2 crossover points is around that point. However, if the value exceeds 2, it indicates multiple groups of crossover points around the point.

## Data Records

The “Tibetan Plateau ICESat-2 Crossover Points Dataset” can be found in CSV and SHP formats at the National Tibetan Plateau Data Center (10.11888/RemoteSen.tpdc.300749)^32^. This dataset includes all crossover points on the Tibetan Plateau. The CSV files are suitable for data extraction and analysis, while the SHP files are useful for data visualization.

**Fig. 4:**
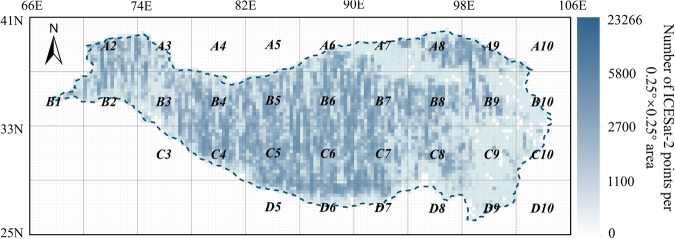
This figure represents the Data Selection Reference Chart for the “ICESat-2 crossover points dataset”. It includes a density map as a background image, which illustrates the number and spatial distribution of crossover points. The boundary of the Tibetan Plateau is indicated by the blue dashed line. Labels such as A2, A3, and D10 specify the regions contained in each file within the dataset.

We’ve arranged the crossover points in the Tibetan Plateau into files labeled A2, A3, A4, …, D10 (Fig. 4) according to their latitude and longitude for easier access. There are two folders, one containing the ATL06 crossover points and the other the ATL08 crossover points. Both folders include SHP and CSV files.

The ICESat-2 crossover points file, which contains ATL06 and ATL08 crossover points, includes the following data columns: the first column (number) represents the group number of the ICESat-2 crossover points; the second (Lon) and third (Lat) columns represent the longitude and latitude of each point; the fourth column (H) displays the elevation information, based on the geodesic height of the WGS84 Ellipsoid (the same as in the ICESat-2 ATL06 and ATL08 source files); the fifth column (Time) shows the time each point was acquired, with data converted from GPS seconds in the ICESat-2 raw data to a date; the sixth column (Ds) displays the distance in meters between two points in each group of crossover points; the seventh column (Dh) represents the elevation difference in meters between the two points in each group of crossover points; and the eighth column (Around_PT) lists the number of points within a 4 m buffer.

The CSV files can be processed with programming languages like Python, C, C++, and MATLAB, and can be read directly as text. The SHP files can be opened with ArcGIS for data visualization and filtering.

**Fig. 5:**
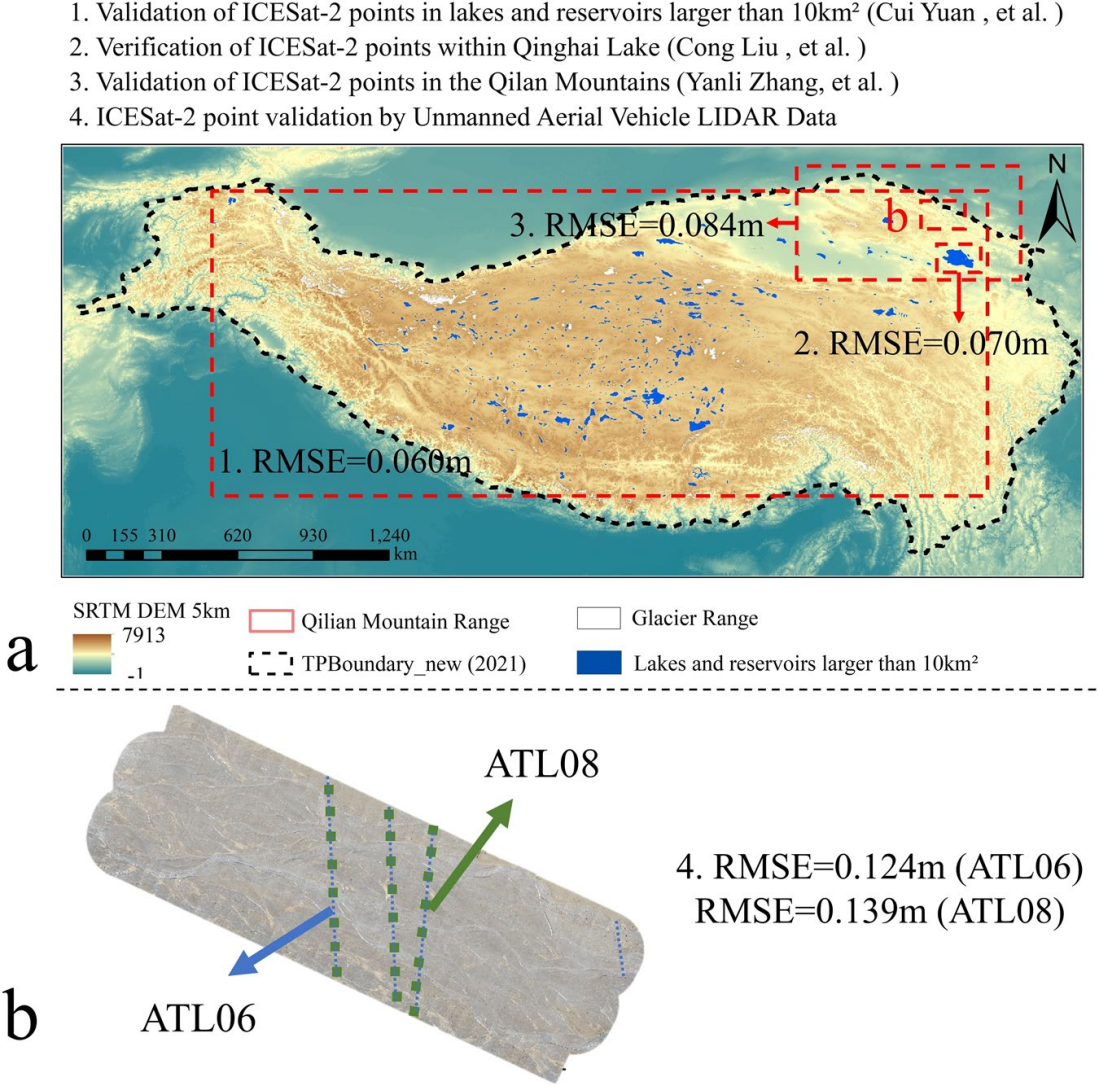
ICESat-2 Accuracy verification. This figure depicts the study areas for three ICESat-2 accuracy validation studies conducted on the Tibetan Plateau^35–37^ as shown in Fig. (**a**). Additionally, the results of UVA observations are illustrated in Fig. (**b**). The white area in the image symbolizes the Tibetan Plateau’s glaciers, whereas the blue area represents lakes larger than 10 km².

## Technical Validation

### Accuracy assessment of ICESat-2 crossover points

The accuracy of ICESat-2, which numerous scholars have confirmed to be around 0.1 m, is widely recognized^33,34^. To assess its measurement accuracy on the Tibetan Plateau, we referenced studies that validated ICESat-2 accuracy over lakes and reservoirs larger than 10 km², Qinghai Lake, and Qilian Mountains (Fig. 5)^35–37^. These studies included both land and water bodies. Additionally, we verified the accuracy of ICESat-2 using field-acquired UAV elevation data (Fig. 5). We compared this data from crossover points with existing glacier and lake studies. For the glaciers, we used the mean annual rate of glacier change (Eqs. 5 and 6) for comparison.

5 $$\Delta {\rm{Hi}}=\frac{{\rm{H}}\_{\rm{Time}}2-{\rm{H}}\_{\rm{Time}}1}{{\rm{Time}}2-{\rm{Time}}1}$$

6 $$\Delta {\rm{H}}=\frac{{\sum }_{{\rm{i}}=1}^{{\rm{n}}}\Delta {\rm{Hi}}}{{\rm{n}}}\times 365$$

ΔHi represents the rate of change between two ICESat-2 points in the i-th group of crossover points. H_Time1 and H_Time2 are the elevations of these points, and Time1 and Time2 are their respective times (Time1 being earlier). ΔH represents the rate of change of the glacier’s mean annual elevation in the validation area.

To calculate the RMSE of the elevation difference at ICESat-2 crossover points, the error propagation formula was utilized based on the ICESat-2 RMSE. Additionally, the RMSE of the rate of change in glacier elevation and the RMSE of the change in lake elevation were calculated using the error propagation formula.

7 $${\rm{z}}={{\rm{f}}}_{1}\cdot {{\rm{x}}}_{1}+{{\rm{f}}}_{2}\cdot {{\rm{x}}}_{2}+\cdots \cdots +{{\rm{f}}}_{2}\cdot {{\rm{x}}}_{2}$$

8 $${{\rm{m}}}_{{\rm{z}}}^{2}={{\rm{f}}}_{1}^{2}\cdot {{\rm{m}}}_{{\rm{x}}1}^{2}+{{\rm{f}}}_{2}^{2}\cdot {{\rm{m}}}_{{\rm{x}}2}^{2}+\cdots \cdots +{{\rm{f}}}_{n}^{2}\cdot {{\rm{m}}}_{{\rm{xn}}}^{2}$$

In the equation, *z* is an indirect measurement and *x*1, *x*2…, *xn* are direct measurements independent of each other. *mx*1, *mx*2…, *mxn* represent the RMSE of *x*1, *x*2, …, *xn* respectively, *mz* represents the RMSE of *z*, and *f*1, *f*2, …, *fn* represent constants respectively.

**Fig. 6:**
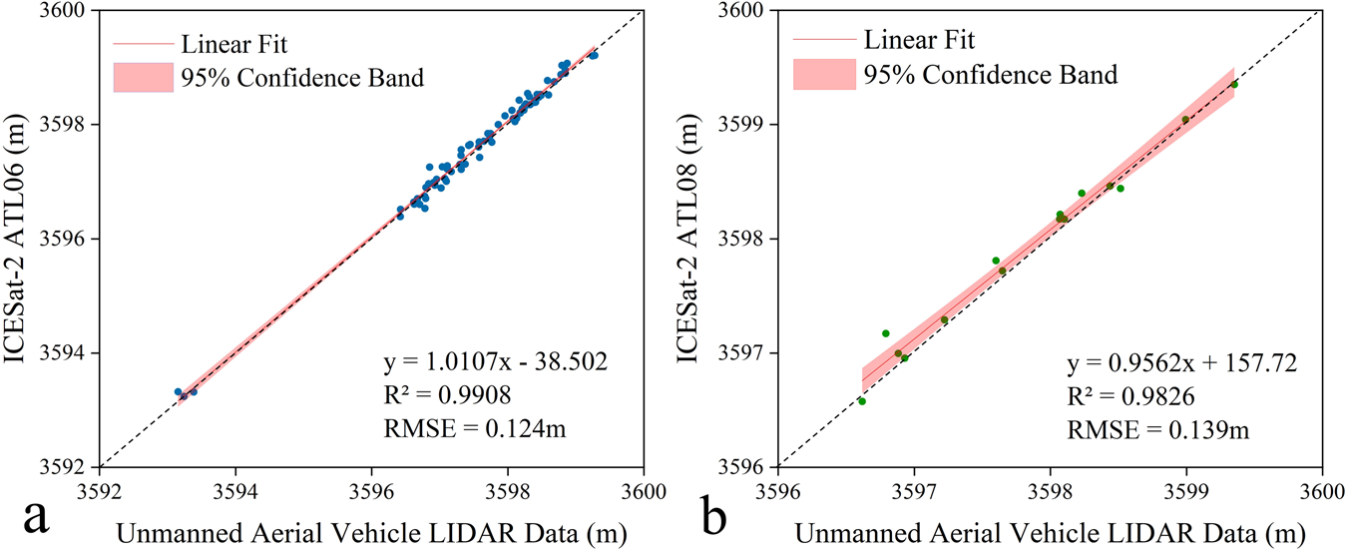
ICESat-2 validation results (using UAV data). Fig. (**a**) shows the relationship between ICESat-2 ATL06 and UAV elevation data. Fig. (**b**) shows the relationship between ICESat-2 ATL08 and UAV elevation data. The fitted equation, RMSE, R², and 95% confidence bands are given in the figure.

### Accuracy Evaluation for ICESat-2 Crossover Points

The validation results of ICESat-2 on the Tibetan Plateau are as follows: 1) Using water level data as the reference, the root mean square error (RMSE) of ICESat-2 on lakes larger than 10 km² is 0.06 m^35^. 2) When using water level station data as the reference, the RMSE of ICESat-2 on Qinghai Lake is 0.07 m^36^. 3) Using ground control points measured by a continuously operating reference system (CORS) as the reference, the RMSE of ICESat-2 on Qilian Mountain is 0.084 m^37^. 4) When using unmanned aerial vehicle (UAV) elevation data as the reference, the RMSE is 0.124 m for ICESat-2 ATL06 and 0.139 m for ICESat-2 ATL08 (Fig. 6). From these four studies, we compute the average of the accuracy results to determine the ICESat-2 accuracy for a single point in the crossover points dataset. The average precision from the four studies is 0.071 m. The elevation difference for each group of crossover points is determined by the difference between the elevations of two ICESat-2 points. As a result, the accuracy of the elevation difference for each group of ICESat-2 crossover points is 0.13 m (Eq. 8).

### Comparison of ICESat-2 crossover points with existing studies

In addition to validating the accuracy of ICESat-2 data, we utilized existing studies to demonstrate the usability of ICESat-2 crossover points data. ICESat-2 produces good measuring results in gentle terrain areas^38^. Most of the glaciers on the Tibetan Plateau are in mountainous areas with high elevation and complex terrain, or in areas that are difficult for humans to access. Moreover, the glaciers on the Tibetan Plateau are more affected by climate change^39^. The lakes on the Tibetan Plateau are evenly distributed, but located in elevation zones that vary greatly, and they experience different situations such as drought and freeze. Therefore, we compared ICESat-2 crossover points on glaciers and lakes with the results of existing studies to explore the usability of ICESat-2 crossover points data for obtaining surface elevation changes on the Tibetan Plateau.

1. **Comparison of ICESat-2 crossover points on glaciers with existing studies**
  We compared the data from Maurer *et al*.‘s “Products of change in mean glacier thickness in the Himalaya (2000–2017)“^40^ and Zeng *et al*.‘s “Qilian Mountains glacier mean annual rate of change.“^41^. Both datasets provide annual change in glacier elevation, and we calculated these rates based on ICESat-2 crossover points. Table 2 provides descriptions of the comparison data and ICESat-2 crossover points.

**Table 2:** Presentation of glacier comparison data.

Validation area	Data	Original data	Time	Data references
Himalayan glacier	Mean glacier thickness variation from multi- sensor DEM	KH-9 Hexagon, ASTER DEM	2000-2017	Maurer, *et al*., 2018
	ICESat-2 crossover point	ICESat-2 ATL06	2019-2022	This article
Qilian Mountains glacier	Mean glacier thickness variation from multi-sensor DEM	ALOS DEM, ICESat-2	2000-2017	Zeng, *et al*., 2023
	ICESat-2 crossover point	ICESat-2 ATL06	2019-2022	This article
  To generate the “Products of change in mean glacier thickness in the Himalaya (2000–2017),” the authors first extracted DEM from KH-9 Hexagon images on 650 glaciers, then fitted the Himalayan glacier elevation on the time series, and finally obtained the changes in glacier elevation, volume, and area. We used this product and ICESat-2 crossover points to calculate the annual mean glacier elevation change separately in parts of the Himalaya and compared the results of the two datasets (Fig. 7b).

**Fig. 7:**
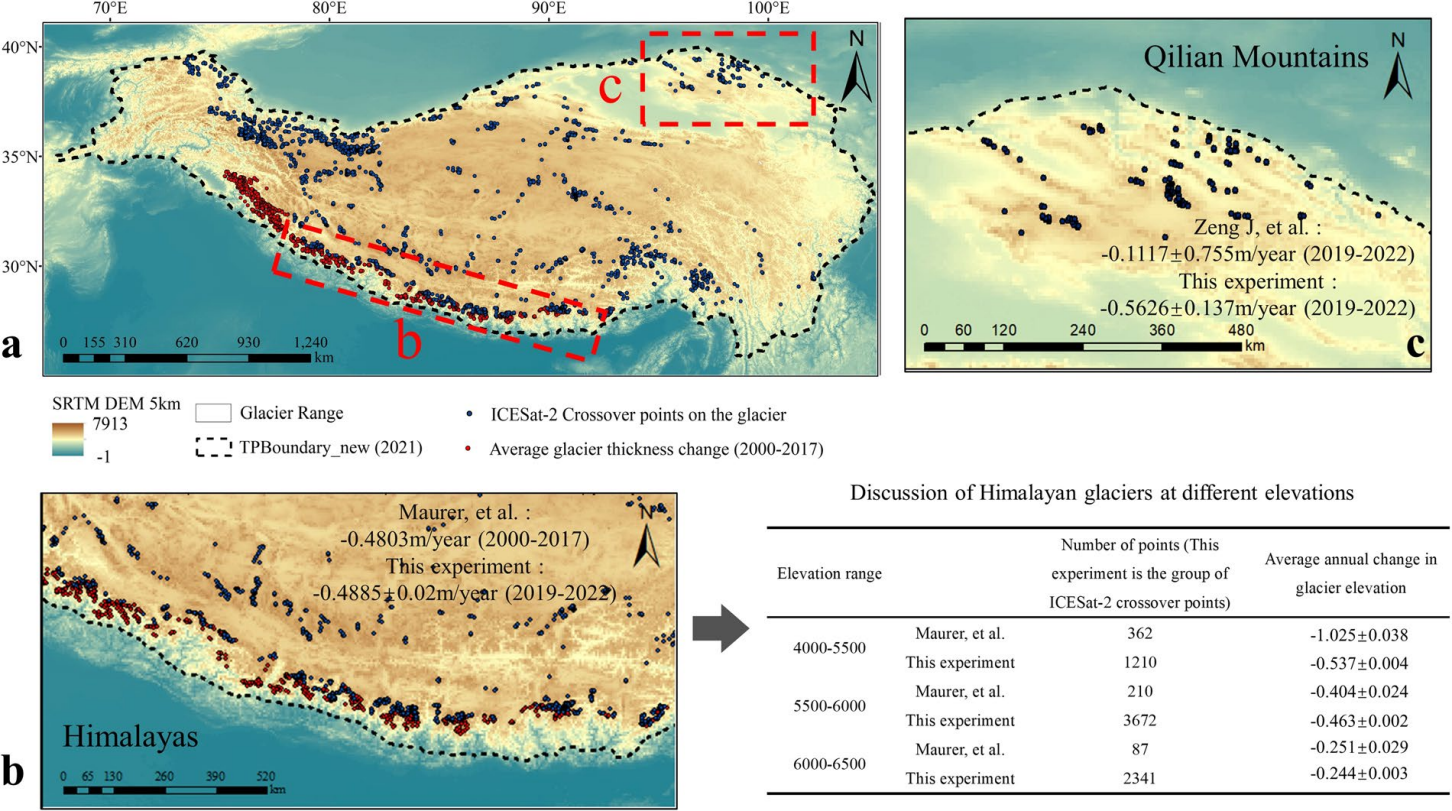
Comparison of ICESat-2 glacier crossover points on the Tibetan Plateau with existing studies. Fig. (**a**) displays glaciers and glacier crossover points on the Tibetan Plateau. The white range represents the Tibetan Plateau glacier boundary, the black dashed line represents the Tibetan Plateau boundary, the blue points represent ICESat-2 crossover points on the Tibetan Plateau glaciers, and the red points represent the Himalayan glacier annual mean change product points. Red boxes indicate comparison areas. Fig. (**b**) shows the Himalayan Glacier Comparison Experiment area, and Fig. (**c**) shows the Qilian Mountain Glacier Comparison Experiment area. The table shows the comparative results of crossover points on Himalayan glaciers according to elevation classification. The elevation zone delineation is based on the DEM of the Himalaya (30 m)^52^.
  The results of the mean annual rate of change of glacier elevation obtained from the “Products of change in mean glacier thickness in the Himalaya (2000–2017)” and the ICESat-2 crossover points data are very similar. From 2000–2017, the mean annual change in glacier elevation obtained from the “Products of change in mean glacier thickness in the Himalaya” is −0.4803 m/year. From 2018–2022, the mean annual change in glacier elevation obtained from ICESat-2 crossover points is 0.4885 ± 0.002 m/year. The difference between the two datasets for the mean annual rate of change of Himalayan glacier elevation is 0.0082 ± 0.002 m/year, which is very small.
  The difference between the results obtained from the two datasets is related to the elevation band. We discussed the differences between the two datasets in elevation bands. There are 3007 groups of ICESat-2 crossover points on the Himalayan glaciers, and the elevations of these crossover points are distributed over the 4000–6500 m elevation band. We divided the comparative experimental area into three elevation bands: 4000–5500 m, 5500–6000 m, and 6000–6500 m. Based on the 30 m digital elevation model of the Himalayas, the number of 4000–5500 m points is less. The difference between the two datasets for the results was 0.488 ± 0.042 m/year, 0.059 ± 0.026 m/year, and 0.007 ± 0.032 m/year in the three elevation bands, respectively. The difference between the results of the two datasets is the largest in the 4000–5500 m elevation band, and the higher the elevation, the smaller the difference between the results. If the “Products of change in mean glacier thickness in the Himalaya” is taken as the true value, it shows that the higher the elevation, the better the results of ICESat-2 crossover points when measuring glaciers.
  It should be noted that ICESat-2 was launched in September 2018, so the rate of change of Himalayan glaciers calculated from ICESat-2 crossover points is for 2018–2022. The “Products of change in mean glacier thickness in the Himalaya” gives the rate of glacier change for the period 2000–2017. There is no overlap in the timing of the two datasets, and the confidence in the comparison results is reduced. Therefore, we used the Qilian Mountain glacier for further comparative experiments.
  This experiment also utilized the rate of glacier change in the Qilian Mountains from existing research and ICESat-2 crossover points for comparison (Fig. 7c). To obtain the rate of glacier change in the Qilian Mountains, the authors corrected the ICESat-2 data using the pyramid registration method, used ICESat-2 and ALOS DEM to obtain the elevation difference, and calculated the elevation change rate of six glaciers within the Qilian Mountains. Both our experiment and this study used ICESat-2 data to obtain glacier elevation. Still, we used the elevation difference of ICESat-2 data in each group of crossover points, while this study used the elevation difference between ICESat-2 and ALOS DEM.
  The mean annual rate of change in glacier elevation in the Qilian Mountains obtained from ICESat-2 crossover points is very close to the results of existing research. We obtained the overall glacier change in the Qilian Mountains by averaging the rates of change in elevation of the six glaciers in the Qilian Mountains from existing research, and the result was −0.1117 ± 0.755 m/year. The mean annual rate of change in glacier elevation in the Qilian Mountains, which we obtained using ICESat-2 crossover points, is −0.5626 ± 0.014 m/year. The results calculated from the ICESat-2 crossover points were within the value range of the existing Qilian Mountains glacier study results.

**Fig. 8:**
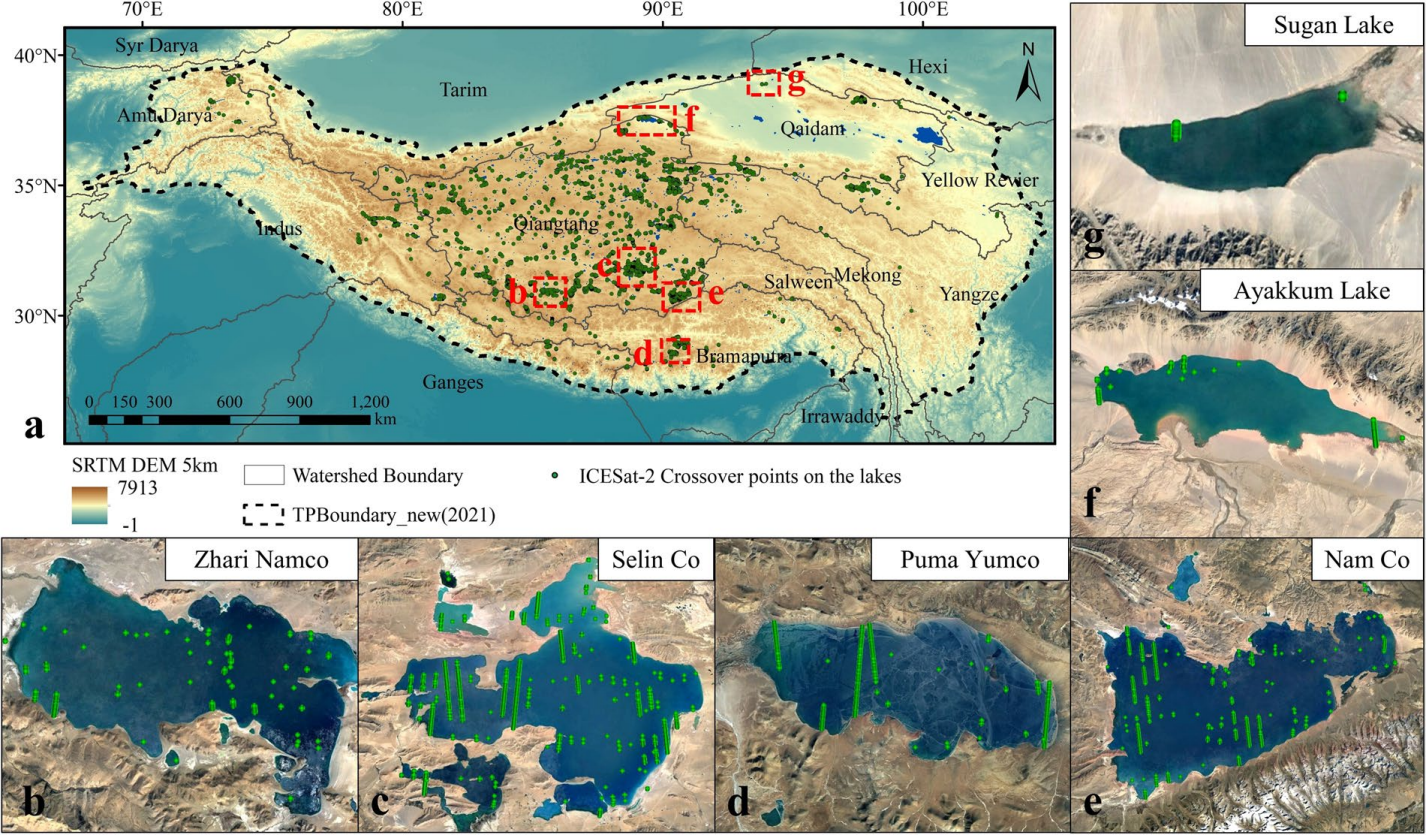
The lakes on the Tibetan Plateau and the ICESat-2 crossover points on those lakes. In Fig. (**a**), the black dashed line shows the boundary of the Tibetan Plateau, the black solid line shows the watershed boundary, the blue area shows the boundary of the Tibetan Plateau lakes, and the red dashed area shows the lakes selected for this comparative experiment. Fig. (**b**) to (**e**) depict the selected lakes and the ICESat-2 crossover points on them.
2. **Comparison of ICESat-2 crossover points on lakes with existing studies**

In our selected lakes, we took into consideration their elevation, freezing conditions, and area.

1. Lakes on the Tibetan Plateau are predominantly found within the elevation range of 2700 m to 5500 m. We classified all Tibetan Plateau lakes into three categories, namely 2700 m-4000 m (with fewer lakes below 4000 m in elevation), 4000 m-4700 m, and 4700 m-5400 m. Among the 2700 m-4000 m lakes, we selected Ayakkum Lake and Sugan Lake. Among the lakes of 4000 m-4700 m, we chose Selin Co and Zhari Namco. Among the lakes of 4700 m-5400 m, we chose Nam Co and Puma Yumco (Fig. 8).
2. These six lakes have varying freezing conditions. Ayakkum Lake has no freezing period, while the edge of Lake Sugan experiences freezing. Selin Co, Zhari Namco, Nam Co, and Puma Yumco have freezing periods. We used this information to determine the applicability of ICESat-2 crossover points in the presence of lake ice.
3. Among the six lakes, Sugan Lake has the smallest area of 128.12 km², Nam Co has the largest area of 2423.39 km², and the remaining four lakes have areas distributed between them. This information was used to determine the applicability of ICESat-2 crossover points on lakes of various areas. The lake information and ICESat-2 crossover points information are presented in Table 3.

**Table 3:** Information of lakes and ICESat-2 crossover points.

Elevation range (m)	Lake Name	Freezing period	Lake area (km²)	Time to verify data (interval time for each set of elevation changes)	Sources of acquisition of lake datasets
2700-4000	Ayakkum Lake	No icing period	1099.28	I:2019/5/2-2020/10/29 II2019/4/29-2019/7/29	Sentinel-3 CryoSat-2
	Sugan Lake	Ice formation at the edges	128.12	I:2018/12/6-2020/3/4	CryoSat-2
4000-4700	Selin Co	With icing period	2426.39	I:2019/4/29-2019/7/29 II:2020/10/5-2021/7/5	Sentinel-3 CryoSat-2
	Zhari Namco	With icing period	1045.9	I:2019/10/21-2020/7/21 II:2019/9/12-2019/11/13	Jason-3
4700-5400	Nam Co	With icing period	2019.61	I:2019/1/30-2019/4/30 II:2019/4/21-2020/12/27	Sentinel-3 CryoSat-2
	Puma Yumco	With icing period	293.38	I:2019/6/2-2020/8/30 II:2019/9/30-2021/3/28	CryoSat-2

Note: The source of lake area is “The lakes larger than 1k m^2^ in Tibetan Plateau (v3.1) (1970s-2022)”^26–28^, dates underlined are within the lake freezing period.

We chose the high-resolution dataset of lake level changes on the Tibetan Plateau from 2002 to 2021 as the comparison data for the ICESat-2 crossover points^42^. The dataset uses eight satellites to fit the elevation changes of lakes larger than 1 km² on the Tibetan Plateau. We selected the crossover points at one- or two-time intervals on each lake (Table 3), and then used the lake elevations provided in the dataset to obtain the elevation change of the lake for each group of time intervals. Finally, we compared the results with those of the ICESat-2 crossover points. The comparative results are shown in Table 4.

**Table 4:** Comparation of crossover points on each lake.

Lake name	group number	ICESat-2 crossover points DH (m)	Number of ICESat-2 points (Number of groups)	QTP lake level dataset DH (m)	Differences between the two DHs (m)
Ayakkum Lake	I	0.6510 ± 0.013	59	0.7054	−0.0544
	II	0.2899 ± 0.016	36	0.3492	−0.0688
Sugan Lake	I	0.5409 ± 0.009	108	0. 3857	0.1552
Selin Co	I	0.0798 ± 0.006	250	0.0596	0.0202
	II	−0.2248 ± 0.004	616	−0.3259	0.1011
Zhari Namco	I	−0.2922 ± 0.020	26	−0.1118	−0.1804
	II	−0.1080 ± 0.024	16	−0.0199	−0.0881
Nam Co	I	0.0178 ± 0.004	655	0.0902	−0.0724
	II	0.2597 ± 0.002	1767	0.4771	−0.2174
Puma Yumco	I	0.3705 ± 0.006	270	0.4348	−0.0643
	II	−0.5558 ± 0.031	10	−0.7233	0.1675

Notes. The errors in “ICESat-2 crossover points DH (m)” for each group of experiments are small (mostly less than 0.01 m). In order to facilitate the analysis of the result, the effect of the error was excluded in the calculation of “Differences between the two DHs (m).”

**Fig. 9:**
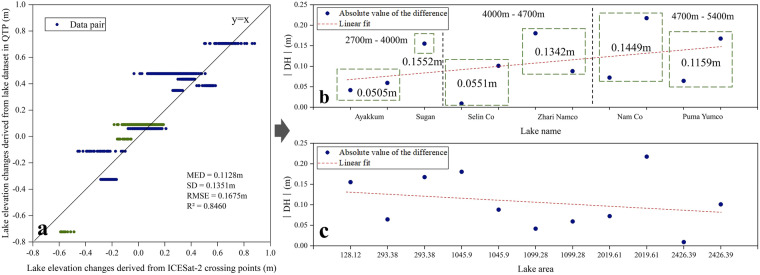
Comparison of ICESat-2 lake crossover points on the Tibetan Plateau with existing studies. Fig. (**a**) displays the elevation changes obtained from the ICESat-2 crossover points and the High-resolution dataset of lake level changes on the Tibetan Plateau from 2002 to 2021. The green points represent the comparison results, including the freezing period, while the black solid line indicates y = x. Fig. (**b**) shows the relationship between the absolute difference between the two data sets (we used these two data sets to calculate the lake elevation change separately, and the absolute value of their difference was defined as the absolute difference) and the lake elevation. Fig. (c) shows the relationship between the absolute difference between the two data sets and the lake area. The trend line is represented by the red dashed line.

There is a strong correlation between the ICESat-2 crossover points and the elevation changes obtained from the high-resolution dataset of lake level changes on the Tibetan Plateau from 2002 to 2021 (Fig. 9a). In the eleven groups of compared data, the absolute difference between the results obtained from the two datasets was a minimum of 0.009 m and a maximum of 0.2174 m, with an RMSE of 0.1675 m. The results of the two datasets in calculating the lake elevation change are very similar. Furthermore, the R² value of the two datasets is 0.8460, indicating a strong correlation. Therefore, it is feasible to calculate lake elevation changes using ICESat-2 crossover points data.

In addition, during the freezing period, the absolute differences between the three groups of data were 0.1675 m, 0.0724 m, and 0.0881 m. It was observed that lake ice did not affect the measurements at the ICESat-2 crossover points.

Lake elevation and area impact the absolute difference between the two datasets. (1) The higher the lake elevation, the larger the absolute difference between the two datasets (Fig. 9b). Among the six lakes, Ayakkum Lake has the lowest elevation (around 3800 m), and the mean value of the absolute difference between the two datasets within the lake is 0.0505 m. Puma Yumco has the highest elevation (around 4980 m), and the mean value of the absolute difference between the two datasets in the lake is 0.1159 m. The mean values of the absolute differences of the two datasets in the four lakes of Sugan Lake, Selin Co, Zhari Namco, and Nam Co are 0.1552 m, 0.0551 m, 0.1342 m, and 0.1449 m, respectively. The absolute difference between the two datasets shows an increasing trend with increasing lake elevation.

## Usage Notes

### Advantages of the ICESat-2 crossover points dataset on the Tibetan Plateau

The ICESat-2 crossover point data can be used in two ways. Firstly, it can be used as elevation difference data. The ICESat-2 crossover point data provides elevation differences before and after a specific time. The RMSE of the elevation difference provided by each group of ICESat-2 crossover points is 0.322 m. The crossover point datasets can give surface elevation changes on a point scale with high accuracy. The existing applications of ICESat-2 include obtaining changes in water bodies, land vegetation, glaciers, and terrain, generating DEM, and using it as validation data. However, no dataset provides surface elevation changes over the entire Tibetan Plateau. The elevation difference on point scales given in the crossover points dataset can provide a reference for surface changes on the Tibetan Plateau.

The high spatial resolution and rich amount of data in the ICESat-2 crossover points can provide more terrain change details. The diameter of the laser footprint of ICESat-2 is 17 m, which is much smaller than the other satellites, such as Envisat, CryoSat-2, Jason-3, and Sentinel-2A. Smaller laser footprint diameters reduce errors due to slopes or complex terrain. Some scholars use the elevation of all points along the track direction to get the surface elevation change in that direction^43^. This provides a method for us to understand the terrain of inaccessible areas. The ICESat-2 crossover points data gives the elevation difference on the point scale, and the density of points on the Tibetan Plateau reaches 2.050 group/km², so the crossover points dataset can provide more terrain details for the surface changes on the Tibetan Plateau.

**Fig. 10:**
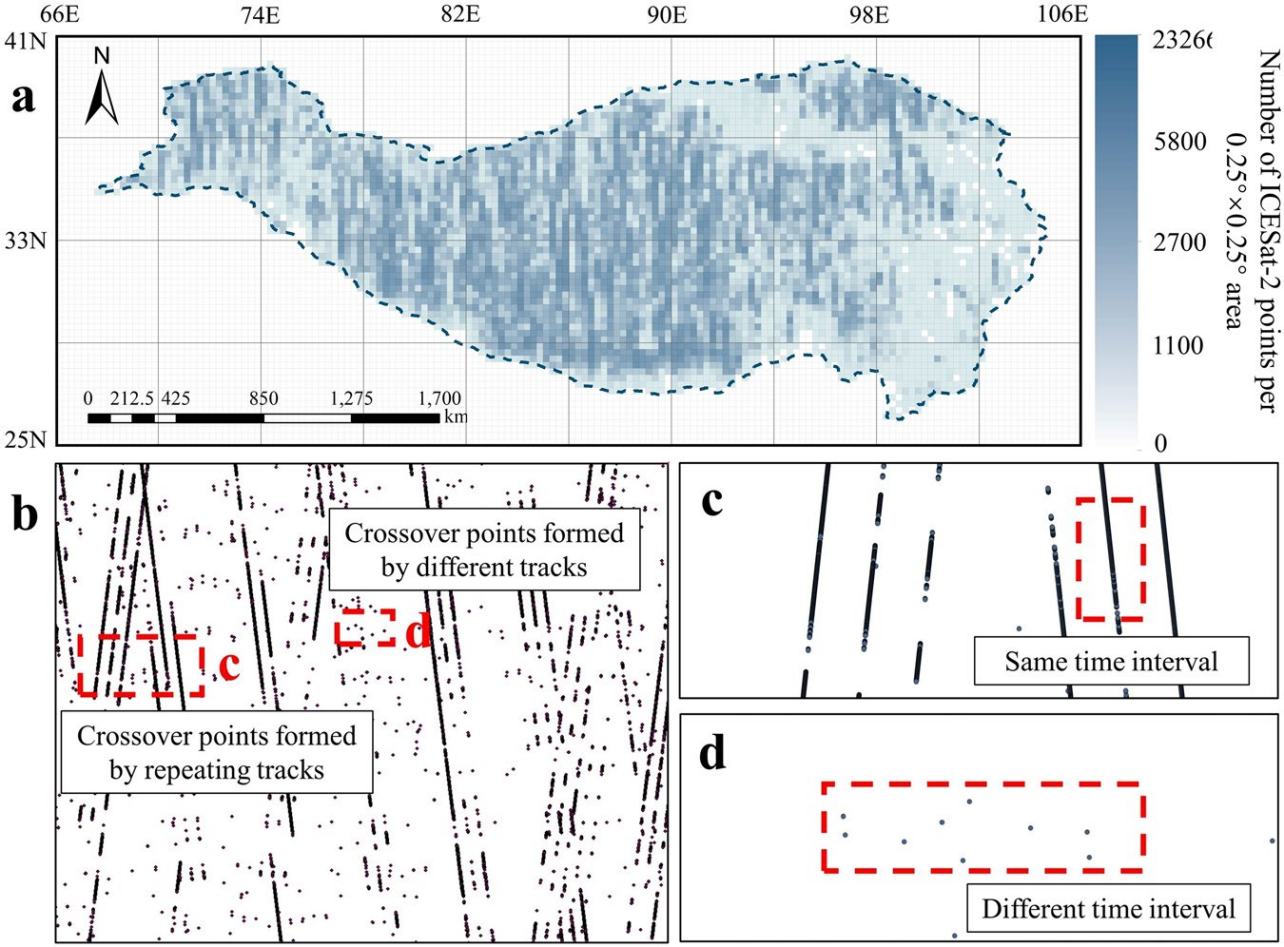
Spatial distribution characteristics of the two types of ICESat-2 crossover points (Fig. 2a). Fig. (**a**) displays a point density map of ICESat-2 crossover points on the Tibetan Plateau. Fig. (**b**) shows ICESat-2 crossover points in a region of Fig. (**a**). Fig. (**c**) provides a schematic representation of crossover points determined by repeating orbits, while Fig. (**d**) shows crossover points determined by different tracks.

There are two types of ICESat-2 crossover points. The first type involves continuous crossover points determined by repeated tracks, while the second type involves discrete crossover points determined by different tracks (Fig. 10). Crossover points determined by repeating orbits have the characteristic of appearing continuously in space in many groups with the same time interval in the direction of the track (Fig. 10c). This improves the accuracy of surface changes over the same time interval. On the other hand, crossover points determined by different tracks are more discrete (Fig. 10d), and each group of crossover points has different time intervals. This improves the time coverage of ICESat-2 crossover points.

ICESat-2 data are commonly used to measure elevation changes. However, crossover point datasets offer unique values in some scenarios. Firstly, they can reveal subtle features of various terrain elevation changes, such as those in glaciers, permafrost, forests, and lakes. These features might not be apparent in the overall change of the Tibetan Plateau, but they are vital for studying environmental changes in specific areas. They can shed light on phenomena like terrain collapse caused by permafrost melting, glacier collapse within large glaciers, and lake disappearance due to extreme climate.

Secondly, crossover point datasets can enhance many existing models of the Tibetan Plateau. For instance, they can refine hydrological models by including elevation changes of glaciers or permafrost, and provide additional validation data for terrain models.

Moreover, crossover point datasets supplement, rather than replace, large-scale elevation change datasets. They offer detailed information that the large-scale datasets might miss. By combining the two, a more comprehensive data system can be created.

**Fig. 11:**
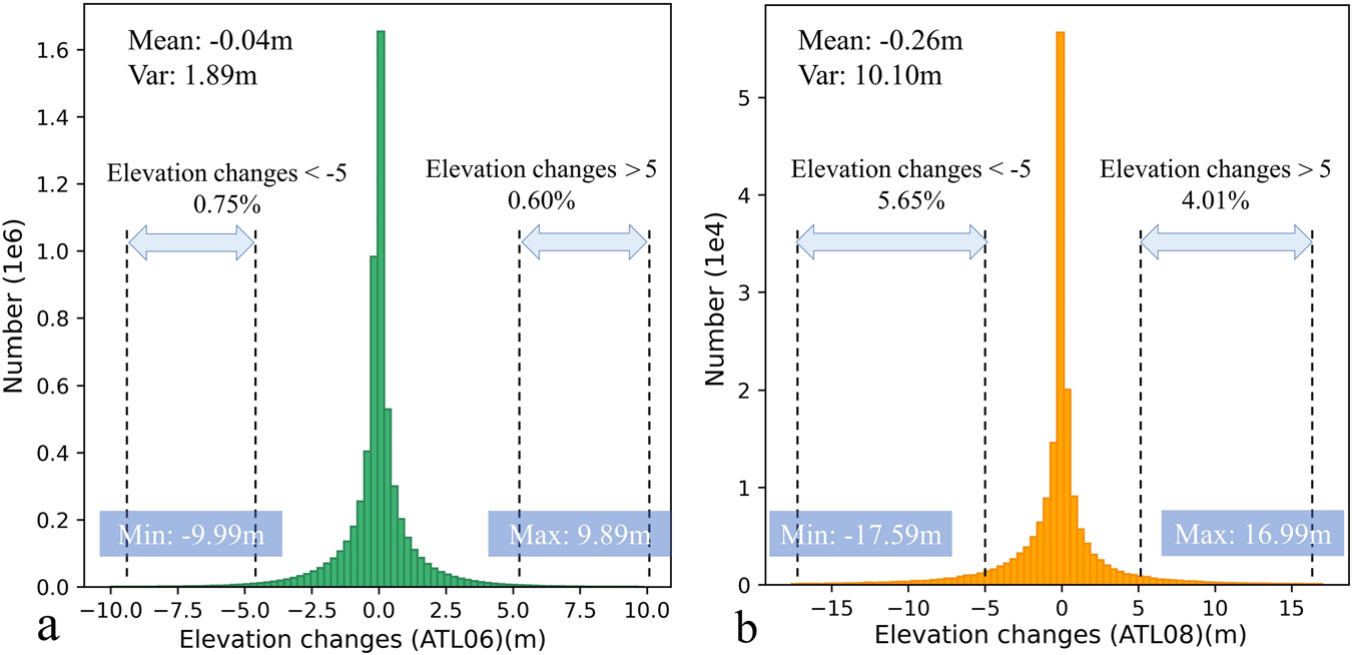
Statistical measures of elevation changes at crossover points. Fig. (**a**) displays a histogram of elevation changes determined by ATL06 at the crossover points. Fig (**b**) presents a histogram of elevation changes determined by ATL08 at the crossover points.

### Statistical measures of elevation changes at crossover points

The elevation difference of the crossover points follows a normal distribution (Fig. 11). According to the ICESat-2 ATL06 product, the elevation changes range from −9.99 m to 9.89 m, averaging −0.04 m with a variance of 1.87 m. On the other hand, the ATL08 product shows a broader range from −17.59 m to 16.99 m, averaging −0.26 m with a variance of 10.10 m². The ATL08 product has a noticeably larger variance than the ATL06, and its data surpasses ATL06 in both maximum and minimum values. This further confirms the greater dispersion of the ATL08 data in elevation difference.

We chose the ATL08 product to supplement the areas of the Tibetan Plateau, namely the eastern and western regions, not covered by the ATL06 product. This region encompasses the Tarim Basin, the Hengduan Mountains, and the Pamir Plateau. The Tarim Basin includes a vast desert area with significant southward dune movement^44^. The Hengduan Mountains are characterized by hazardous terrain with intersecting mountains and rivers, as well as diverse vegetation^45^. The ATL08 product’s measurements of elevation changes in surface and vegetation in these areas can be affected by seasonal vegetation, desert alterations, and terrain relief. This could lead to more significant fluctuations in elevation at ATL08 crossover points.

The number of crossover points determined by ATL08 is less than that determined by ATL06. We selected a 10,000 km² area in the central Tibetan Plateau for comparison. Within this area, the density of crossover points determined by ATL06 is 3.61 groups/km², whereas the density determined by ATL08 is 0.08 groups/km². Given that ATL06 has a spatial resolution of 20 m and ATL08 has a resolution of 100 m, the ATL06 product has more laser points in the same area, leading to more data on crossover points.

### Factors affecting the accuracy of crossover points

The accuracy of elevation differences obtained from ICESat-2 crossover points is influenced by several factors. In this experiment, we compared the results of crossover points with existing studies on glaciers and lakes. We discovered that the accuracy of ICESat-2 crossover points on glaciers increases with elevation, while the accuracy of ICESat-2 crossover points on lakes decreases with elevation. The relationship between the accuracy of ICESat-2 crossover points and elevation varies for different surface types. Additionally, the accuracy of ICESat-2 crossover points on lakes increases with lake area. This is because larger lakes have more crossover points, resulting in a smaller chance for error.

Slope may also affect the accuracy of ICESat-2 crossover points. The overall slope of the Tibetan Plateau ranges from 0 to 54 degrees, with the majority of slopes concentrated in the 0 to 50-degree range^46,47^. Some scholars have analyzed slope in relation to ICESat-2 ATL06 accuracy using CORS and UAV data in the Qilian Mountains. The results showed that while slope affects ICESat-2 ATL06, ICESat-2 improves the impact of terrain on the data by increasing the sampling frequency and crossover measurements^37^. In this experiment, the ICESat-2 crossover points dataset was not filtered for slopes to ensure an adequate amount of data. However, in future research, we can filter the crossover points dataset using slope depending on the specific research objectives.

### Applicable scenarios for using the ICESat-2 crossover points dataset

The ICESat-2 crossover points dataset offers a new method for obtaining surface elevation changes on the Tibetan Plateau. This dataset is a high spatial resolution surface elevation change data produced based on discrete ICESat-2 ATL06 and ATL08 points. Each group of crossover points gives the amount of change in elevation before and after a period of time over an area of about 17 m in diameter. So, this dataset can be used to obtain elevation changes of finer surface details on the plateau. Here are some ideas for using ICESat-2 crossover points data:

1. The Tibetan Plateau has the world’s largest area of perennial permafrost at low and middle latitudes. Climate change has led to the degradation of this permafrost, threatening infrastructure security. One study showed that by 2050, about 38 percent of roads, 39 percent of railways, 39 percent of power lines, and 21 percent of buildings will be exposed to high-risk areas^48^. To address this issue, we can use ICESat-2 crossover points data to obtain permafrost surface elevation changes in the corresponding areas (such as along railways and highways) and understand intra- and inter-annual changes of permafrost. Moreover, drastic small-scale surface deformation often occurs in permafrost regions. This includes surface fractures, subsidence, and landslides caused by melting permafrost^49,50^. Therefore, the ICESat-2 crossover points data can also be used as validation data for other permafrost active layer change studies.
2. Glaciers on the Tibetan Plateau are abundant, but their changes have intensified in recent years. Some studies showed that a quarter of the glaciers have melted in the last forty years, which could harm the climate and people. To address this issue, we can use ICESat-2 crossover points data to obtain inter- and intra-annual glacier changes. In addition, ICESat-2 crossover points data can also refine existing studies of glacier elevation change. Although existing glacier studies on the Tibetan Plateau can obtain large-scale glacier changes, finer elevation changes that address surface details are still needed. ICESat-2 crossover points data can be used as supplementary data for large-scale glacier studies, supplementing internal details of large glaciers and elevation changes of small glaciers.
3. Combining ICESat-2 crossover points data with multi-source remote sensing data. In the ICESat-2 crossover points data, each group of crossover points is spatially and temporally discretely distributed without forming a continuous time series, so it cannot get an area’s continuous surface elevation change directly. We can merge Envisat, CryoSat-2, Jason-3, and Sentinel-3A data with ICESat-2 crossover points to obtain elevation changes in a continuous time series. The spatial resolution and acquisition method of each type of data is different. Therefore, this method may obtain better results in areas with flatter terrain, such as large lakes, deserts, or permafrost with lower slopes. Additionally, the multiple groups of crossover points extracted from this experiment in a specific area can provide more data for studying time series changes in elevation.
4. As validation data. Firstly, ICESat-2 crossover points data can be used to validate surface elevation changes on the Tibetan Plateau. Secondly, since the terrain of the Tibetan Plateau is complex, the ICESat-2 crossover points data can be used as validation data for many inaccessible areas when it is used as a single point. Compared with the existing DEM data, ICESat-2 crossover points data have higher accuracy, and each point elevation has acquisition time. When validating the existing data with the help of ICESat-2 crossover points, averaging the spatially neighboring ICESat-2 crossover points can remove accidental errors if we do not consider the acquisition time. The crossover points data, which cover the entire Tibetan Plateau, can significantly contribute to the study of surface elevation there. For instance, they can be used to verify elevation changes in mountains and deserts, areas typically inaccessible to humans.
5. Combining ICESat-2 crossover points data with various models. Since ICESat-2 ATL06 is the product for land ice heights, the ATL06 crossover points dataset does not cover the Qaidam Basin and the eastern part of the Tibetan Plateau. We supplemented the areas with missing ATL06 data. The final crossover points now include a variety of terrain types across the Tibetan Plateau region, such as glaciers, permafrost, lakes, deserts, and forests. The advancements in machine learning have enabled the use of high-precision discrete surface elevation changes as a rich sample set. This allows for the attainment of higher-resolution surface elevation changes across the Tibetan Plateau. The development of models for glaciers, snowpack, hydrology, ecology, and vegetation on the Tibetan Plateau also would benefit from this. Crossover point datasets can provide ample validation data and serve as input data for these models.

The ICESat-2 satellite is still operational, and we will continue to update the crossover points dataset with more ICESat-2 data volume, resulting in more crossover points. As ICESat-2 data grows, the occurrence of multiple crossover point groups within a certain range will increase and be collected, with updates in future dataset versions.

## Data Availability

The script used to process the ICESat-2 data and extract ICESat-2 crossover points from it is available at the following link: https://github.com/snowhydro/icesat-cross-point.
